# The Effect of Total Hip Arthroplasty on Sports and Work Participation: A Systematic Review and Meta-Analysis

**DOI:** 10.1007/s40279-018-0924-2

**Published:** 2018-04-24

**Authors:** Alexander Hoorntje, Kim Y. Janssen, Stefan B. T. Bolder, Koen L. M. Koenraadt, Joost G. Daams, Leendert Blankevoort, Gino M. M. J. Kerkhoffs, P. Paul F. M. Kuijer

**Affiliations:** 1 Department of Orthopaedic Surgery, Academic Medical Center, Amsterdam Movement Sciences, University of Amsterdam, Meibergdreef 9, 1105 AZ Amsterdam, The Netherlands; 2 Department of Orthopaedic Surgery, Foundation FORCE (Foundation for Orthopaedic Research Care and Education), Amphia Hospital, Molengracht 21, 4818 CK Breda, The Netherlands; 3 Academic Center for Evidence-Based Sports Medicine (ACES), Meibergdreef 9, 1105 AZ Amsterdam, The Netherlands; 4 Medical Library, Academic Medical Center, University of Amsterdam, Meibergdreef 9, 1105 AZ Amsterdam, The Netherlands; 5 Coronel Institute of Occupational Health, Amsterdam Public Health Research Institute, Academic Medical Center, University of Amsterdam, Meibergdreef 9, 1105 AZ Amsterdam, The Netherlands

## Abstract

**Background:**

Total hip arthroplasty (THA) is a successful procedure to treat end-stage hip osteoarthritis. The procedure is increasingly performed in adults of working age, who often wish to return to sports (RTS) and return to work (RTW). However, a systematic overview of the evidence on RTS and RTW after THA is lacking.

**Objectives:**

Our aim was to systematically review (1) the extent to which patients RTS and RTW after THA, including (2) the time to RTS and RTW.

**Methods:**

We searched MEDLINE and Embase from inception until October 2017. Two authors screened and extracted the data, including study information, patient demographics, rehabilitation protocols and pre- and postoperative sports and work participation. Methodological quality was assessed using the Newcastle–Ottawa scale. Data on pre- and postoperative sports and work participation were pooled using descriptive statistics.

**Results:**

A total of 37 studies were included, of which seven were prospective studies and 30 were retrospective studies. Methodological quality was high in 11 studies, moderate in 16 studies, and low in ten studies. RTS was reported in 14 studies. Mean RTS was 104% to the pre-surgery level and 82% to the pre-symptomatic sports level. Time to RTS varied from 16 to 28 weeks. RTW was reported in 23 studies; the mean was 69%. Time to RTW varied from 1 to 17 weeks.

**Conclusion:**

A great majority of patients RTS and RTW after THA within a timeframe of 28 and 17 weeks, respectively. For the increasingly younger THA population, this is valuable information that can be used in the preoperative shared decision-making process.

**Electronic supplementary material:**

The online version of this article (10.1007/s40279-018-0924-2) contains supplementary material, which is available to authorized users.

## Key Points

**Table:** 

Eight out of ten patients return to a sports level equal to their pre-symptomatic level after total hip arthroplasty. A return to high-impact sports activities is less likely but is definitely possible in experienced patients.
Overall, seven out of ten patients return to work after total hip arthroplasty. However, modern-day studies showed a mean return to work of 86%. This might be attributed to the increase in total hip arthroplasty in patients aged < 65 years as well as more liberal work recommendations.
Preoperative sports participation and lower age are predictive of a successful return to sports. Preoperative sick leave and a high workload are predictive of no return to work.

## Introduction

Total hip arthroplasty (THA) to treat severe osteoarthritis (OA) of the hip joint is one of the most successful orthopedic procedures performed nowadays [1]. The use of THA has skyrocketed in recent decades. In the USA, utilization rates of THA doubled from 102 to 210 per 100,000 between 2000 and 2011 [2, 3]. Likewise, THA utilization rates have been steadily increasing in other developed countries, including the UK, Australia, and most European countries [2]. Numerous reasons for the increasing incidence of THA exist, including the ageing society and the growing prevalence of obesity [4]. Another important factor is patients’ participation in an active lifestyle and in highly demanding work and sports activities, both at younger and at older ages [5]. The largest increase in absolute numbers of THA is observed in patients aged < 65 years [2, 6], with the greatest percentage increase in the group aged 45–54 years [3].

There is patient demand for improved recovery after hip arthroplasty. Alterations of the technique with tissue-sparing approaches may improve early recovery [7]. Improved bearing materials have shown better outcomes with less wear problems, allowing a return to daily activities with full impact [8]. In a younger and more active patient population, a return to daily activities includes return to sports (RTS) and return to work (RTW) [9, 10]. Although of major importance to the patient, scientific data on RTS and RTW issues after THA have been scarce. A systematic review concerning RTW identified seven studies and found that RTW ranged from 25 to 95% at 1–12 months postoperatively [11]. Timing of RTW ranged from 1.1 to 13.9 weeks. However, the authors did not pool their data for RTW because of the heterogeneity of included studies and found that the overall methodological quality of the included studies was moderate to low [11]. Since the search for the abovementioned review in 2013, newer studies have also focused on RTW after THA [12, 13].

No previous study has systematically summarized the available evidence on RTS after THA. Klein et al. [14] evaluated the allowable or recommended sporting activities after THA based on recommendations from 549 orthopedic surgeons. In general, low-impact activities such as swimming, walking, and dancing were allowed, and intermediate-impact sports such as Pilates, ice-skating, and downhill skiing were allowed in individuals experienced in these activities. There was consensus between the surgeons that high-impact sports were not allowed. However, those recommendations were based on expert opinion and not supported by results from clinical studies. Although some research has been performed on RTS after THA, the actual extent of and time to RTS remain largely unknown [15, 16]. Furthermore, there appears to be a discrepancy between previous RTS recommendations and the actual sports participation that is achieved by modern-day THA patients [17]. Lastly, prognostic factors for RTS after THA have never been systematically reviewed.

A comprehensive evidenced-based review of the literature on the possibility of returning to both sports and work after THA is lacking. Therefore, the purpose of the present analysis was to systematically review the available evidence on the extent to which THA patients RTS and RTW and the timing of this return. In addition, reported prognostic factors for RTS and RTW were reviewed. The results of this study may aid the orthopedic surgeon in providing adequate guidance to future patients about the likelihood of RTS and RTW after THA.

## Methods

### Search Strategy

The PRISMA (Preferred Reporting Items for Systematic reviews and Meta-Analyses) guidelines were used for this systematic review [18]. A research protocol was developed before the literature search was commenced. This protocol was published online at the PROSPERO International prospective register of systematic reviews (http://www.crd.york.ac.uk/PROSPERO/; registration number CRD42016052471). A clinical librarian (JD) developed the search strategy in close cooperation with the first author. A systematic search in the MEDLINE and Embase databases was performed from inception until 24 October 2017. A scoping search consisting of citation analysis identified a set of relevant references. From this reference set, search concepts and, subsequently, search terms were derived. Main concepts were (sport OR work-related activities) AND {[(recovery of function OR surgery) AND longitudinal study design] OR (return to sports & work)} AND total hip arthroplasty. Details of the search strategy can be found in the Electronic Supplementary Material (ESM), Appendix S1. The reference lists of selected studies were screened to identify additional studies for inclusion. We also performed a cited reference search in Web of Science to identify more recent studies.

### Eligibility Criteria and Study Selection

The results of our MEDLINE and Embase searches were cross-checked and duplicate papers were excluded. The titles and abstracts of the remaining papers were screened by two independent reviewers (AH, KJ) for suitability for inclusion. The Rayyan screening tool for systematic reviews was used to screen titles and abstracts [19]. Discrepancies were resolved by discussion; where there was doubt, the article was included in the full-text screening process. One author (KJ) then selected suitable studies based on the eligibility criteria established in the research protocol. This selection was then reviewed by a second author (AH), and discrepancies were resolved by discussion or by consulting a third reviewer (PK). Inclusion criteria were as follows: observation or intervention studies, describing patients with hip OA who underwent THA, who were participating in sports or working before surgery and intended to RTS and/or RTW after surgery. No restrictions were placed on language or publication year. Review articles were excluded for data extraction, but their references were checked for additional studies that were not identified in our primary search. Exclusion criteria were no primary diagnosis of hip OA, hip resurfacing arthroplasty, and no specific data about RTS or RTW.

### Outcome Measures

The primary outcome was the percentage of patients RTS and/or RTW, and the timing of RTS/RTW. The secondary outcomes were specific activity outcome measures, including the University of California Los Angeles (UCLA) activity score (1–10, where 1 = no physical activity and 10 = extremely active) [20] and the Grimby scale (1–6, where 1 = hardly any physical activity and 6 = regular hard exercise) [21]. The Reichsausschuss für Arbeitszeitermittlung (REFA; German workload classification) classification system was used (0 = work with no physical demand and 4 = work with most heavy physical demand) as a work-related outcome measure.

### Data Extraction

Data were systematically extracted from the included studies by one author, and this was independently repeated by a second author. Disagreements were resolved by discussion and, if necessary, by consulting a third reviewer. The authors used a standardized data extraction form that included the following data: (1) study information: author, year, country; (2) study design and follow-up; (3) information about study population: cohort, population size, sex, age, body mass index (BMI), comorbidities; (4) description of rehabilitation protocols used; (5) definition of outcome measures; (6) preoperative activity and definition, e.g., pre-symptomatic or at time of surgery; (7) postoperative activity; (8) RTS and RTW percentages and time to RTS and RTW; (9) confounding factors taken into account in the study, such as sex, age, BMI, motivation, surgeon’s advice, preoperative sports participation, workload or sick leave. When information was missing or unclear, we approached the authors for additional information.

### Quality Assessment

We used the Newcastle–Ottawa Scale (NOS) to assess the methodological quality of the studies [22] (ESM Appendix S2). One author (KJ) reviewed the quality of all included studies. This was independently repeated by two authors (AH and PK), who each reviewed half of the included studies. Discrepancies were resolved by discussion. With the NOS, every study is assessed on eight items, which are subdivided into three groups: the selection of the study groups (four items), the comparability of the groups (one item), and ascertainment of the outcomes of interest for cohort studies (three items). A maximum of one star can be allotted to every item in the selection of the study groups and ascertainment of the outcome groups of interest for cohort studies. A maximum of two stars can be assigned to the comparability of the groups. Thus, a study can receive a maximum of nine stars. In a previous systematic review, Takahashi and Hashizume [23] defined a study as of high quality if it scored seven or more stars. To further improve the distinction between high- and low-quality studies, we defined high-quality studies as those obtaining eight or nine stars for the present systematic review. A study was rated as moderate quality if it obtained six or seven stars. Studies with five stars or fewer were rated as low quality.

### Pooling Data

Patient demographics were analyzed using descriptive statistics. For pooling of RTS and RTW data, we used the methodology previously described by Witjes et al. [24] and Hoorntje et al. [25] for their studies on RTS and RTW after knee arthroplasty and knee osteotomy. Concerning RTS, studies were included if detailed numbers of patients who participated in specific sports pre- and postoperatively were presented. Sports were categorized as low, intermediate, or high impact based on the classification by Vail et al. [26] (ESM Appendix 3). We calculated pooled RTS percentages using descriptive statistics by comparing pooled pre- and postoperative sports participation data. Finally, these results were described as the average number of sports per patient. RTS percentages were analyzed for all included studies and separately for the high-quality studies. RTW data were pooled using descriptive statistics for studies that provided pre- and postoperative numbers of working patients. RTW percentages were analyzed for all included studies and separately for the high-quality studies. RTW was also analyzed separately for studies published before and after 2000 because of the large increase in THA utilization in patients of working age in the last 2 decades [27]. All statistical analyses were performed using SPSS (version 24.0.; IBM Corp, Armonk, NY, USA).

## Results

### Literature Search

**Fig. 1:**
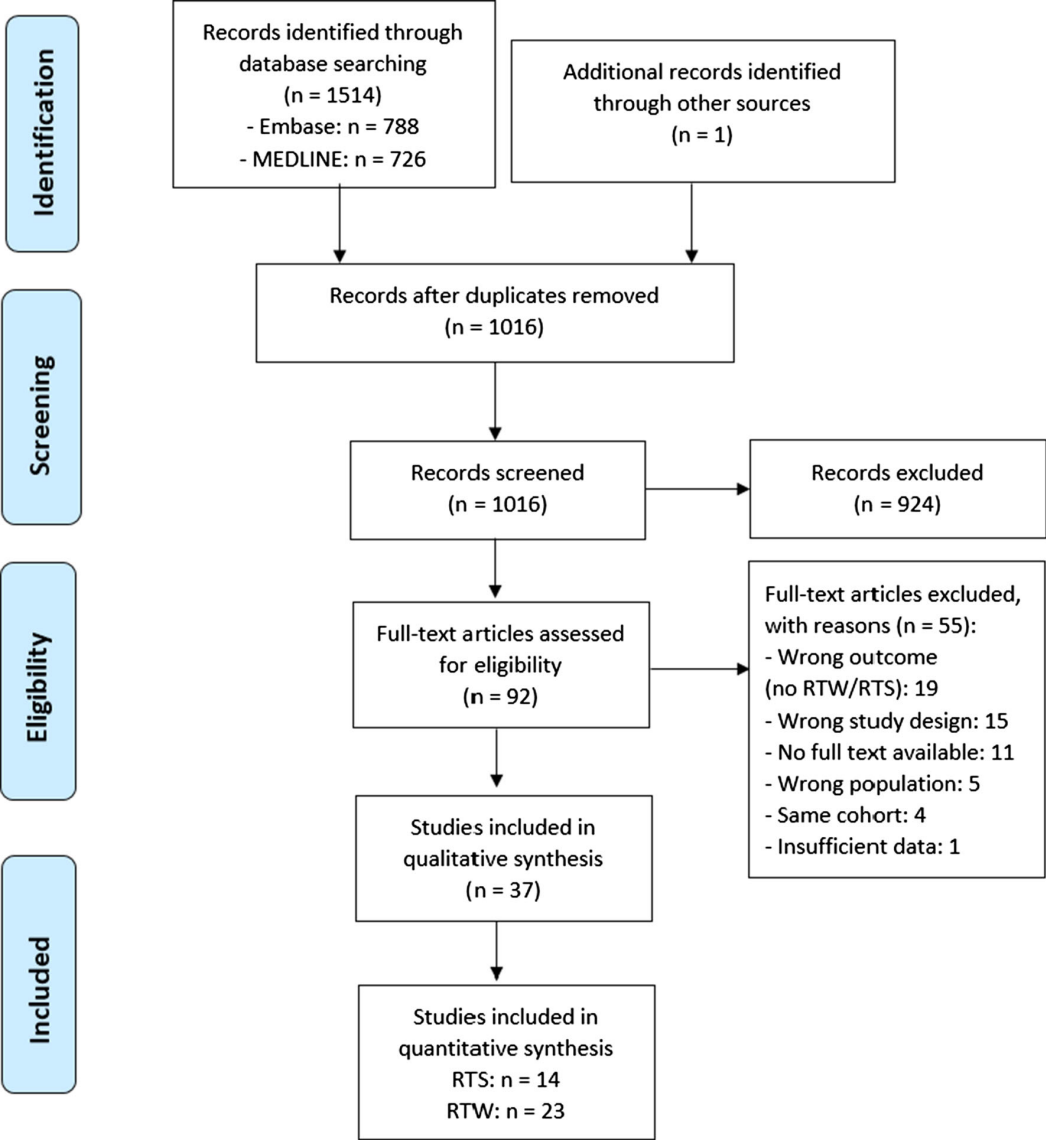
PRISMA (Preferred Reporting Items for Systematic reviews and Meta-Analyses) flow diagram. *RTS* return to sports, *RTW* return to work

Figure 1 presents the PRISMA flowchart of our screening process. Our primary search in the MEDLINE and Embase databases yielded 1514 articles. After removing 498 duplicates, 1016 articles remained. After screening of titles and abstracts, we excluded 924 articles. Thus, 92 full-text articles were screened for eligibility. After full-text screening, 37 studies were included. Reasons for exclusion of full-texts were wrong outcome measure (*n* = 19), wrong study design (*n* = 15), no full text available (*n* = 11), wrong population (*n* = 5), same cohort (*n* = 4), and insufficient data (*n* = 1). Searching the references of the included studies provided one additional study [28].

### Study Characteristics

Table 1 presents the data extracted for each of the included studies. The studies were published between 1965 and 2016. Eight of the 37 studies included were published before 2000. Three case–control studies, one cross-sectional study, six prospective cohort studies, 24 retrospective cohort studies, two non-randomized controlled studies, and one randomized prospective study were included. Most studies were written in English (*n* = 34), one was written in French, and two were written in German. One study was performed in each of Australia, Denmark, Finland, Greece, Italy, and Switzerland; two were performed in each of Canada, France, Japan, Poland, Spain, Sweden, and the Netherlands; five each were performed in Germany and the UK; and seven were performed in the USA. Of these 37 studies, 13 reported RTS [29–41], 22 reported RTW [12, 13, 42–61], and two reported both RTS and RTW [28, 42].

**Table 1:** Return to sports and work after total hip arthroplasty: data extracted from studies included in the review (*n* = 37)

Study	Study design	Study population^a^	Operation type (+ fixation implant)	Rehabilitation protocol
Abe et al. [29] 2014; Japan; level of evidence: III	Case–control; follow-up: 4.8 y (range 2.3–7.8)	608 pts with OA (85 M [14%], 523 F [86%]) Age: 62 y (range 26–98) BMI: 23.2 (range 14.7–34.2) Co: NR	Primary THA: Cemented 107; uncemented 420	Full weight bearing as tolerated from first postop day Sports participation allowed at 6 mo postop (except contact sports)
Arbuthnot et al. [30] 2007; UK; level of evidence: III	Retrospective; follow-up: 7.6 y (range 2.0–20.0)	66 pts with OA who played golf pre-op (sex NR) Age: 70.4 BMI NR Co NR	Primary THA (not otherwise specified)	NR
Atkinson et al. [42] 2010; UK; level of evidence: I	Non-randomized controlled; follow-up: 2.8 y (range 1.0–5.0)	39 pts with bilateral hip OA Group 1: Single-episode bilateral THR (*n* = 18) (11 M [61%], 7 F [39%] Age: 61.9 BMI and Co NR Group 2: Staged bilateral THR (interval 44 wks [16–88]); *n* = 21 (13 M [62%], 8 F [38%]) Age: 63.7 BMI and Co NR	Uncemented THA (anterolateral approach)	NR
Berger et al. [43]; 2004; USA; level of evidence: II	Prospective; follow-up: 0.3 y	100 pts aged 40–75 y of age without previous hip surgery, BMI < 35 (74 M [74%], 26 F [26%]) Age: 56 (range 41–75) BMI: M 26.8 (range 22.5–33.0); F 24.1 (range 20.6–29.1) Co NR	Uncemented THA (minimally invasive two-incision approach) with rapid rehabilitation protocol	Preop pt education meeting Weight bearing as tolerated postop Start activities as soon as tolerated Outpatient physical therapy
Bohm [44] 2010; Canada; level of evidence: II	Prospective; follow-up: 1.0 y	46 pts who had undergone THA and were working preop RTW: *n* = 40 (21 M [53%], 19 F [47%]) Age: 49.9 BMI and Co NR No RTW: *n* = 6 (1 M [17%], 5 F [83%]) Age: 60.3 BMI and Co NR	Primary THA (not otherwise specified)	NR
Chatterji et al. [31] 2004; Australia; level of evidence: III	Retrospective; follow-up: 1.0–2.0 y	216 pts who had undergone THA 1–2 y before the study Age: 67.8 ± 10.2 Sex, BMI, and Co NR	Primary THA (cemented, uncemented, and hybrid)	Postop physiotherapy until hospital discharge No outpatient physiotherapy treatment
Clyde et al. [45] 2013; USA; level of evidence: III	Retrospective cohort; follow-up: 5.2 y (range 1.4–10.4)	43 pts aged ≥ 18 y receiving workers’ compensation at time of THA Primary THA: *n* = 43 (31 M [72%], 12 F [39%]) Age: 55.0 (range 53.5–56.4) BMI: 31.7 (range 30.6–32.8) Co NR	Primary THA (not otherwise specified)	NR
Danielsson [46] 1965; Sweden; level of evidence: II	Prospective; follow-up: 3.5 y (range 1.0–7.0)	30 pts who underwent THA (10 M [33%], 20 F [67%]) Age: 59 (range 32–76) BMI and Co NR	Primary THA (not otherwise specified)	Physiotherapy for 2.5 wks after operation Start increasing weight bearing after 4 wks
Del Piccolo et al. [40] 2016; Italy; level of evidence: III	Retrospective; follow-up: Conventional stem: 4.4 y (range 1.3–6.0); short stem: 4.5 y (range 1.3–5.7)	78 pts aged 18–50 y with OA Conventional stem: *n* = 58^c^ Age: 38.7 (range 18.0–49.0) Sex, BMI and Co NR Short stem: *n* = 20 Age: 39.9 (range 22.0–49.0) Sex, BMI and Co NR	Primary uncemented THA: standard stem 58; short femoral stem 20	Mobilize on day 2 postop Full weight bearing with crutches from wk 4 Crutches for 6–8 wks Low-impact activities from wk 6 postop
Dubs et al. [28] 1983; Switzerland; level of evidence: III	Retrospective cohort; follow-up: 5.8 y (1.0–14.0)	110 M pts (mostly aged ≤ 60) with hip OA Age: 55.4 (range 29.0–68.0) BMI and Co NR	Cemented THA	NR
Hara et al. [32] 2017; Japan; level of evidence: III	Retrospective; follow-up: 5.7 y (range 1.0–16.6)	524 pts with primary OA or secondary OA due to acetabular dysplasia (84 M [16%], 440 F [84%]) Age: 62.9 (range 22.0–86.0) BMI: 22.9 (± 3.3) Co NR	Uncemented THA (posterolateral approach)	Full weight bearing as tolerated with crutches or walker for 3–4 wks Progress to walking without ambulatory aids when pain free
Huch et al. [33] 2005; Germany; level of evidence: II	Prospective cohort; follow-up: 5.0 y	420 pts aged < 76 y with advanced OA (199 M [48%], 221 F [52%]) Age: 60.5 ± 9.7 BM: < 25: 126 (30%); 25 to < 30: 200 (48%); ≥ 30: 94 (22%) Co: diabetes, 31 (7%); hypertension, 190 (45%); gout, 44 (11%)	Primary THA (not otherwise specified)	NR
Innmann et al. [34] 2016; Germany; level of evidence: III	Retrospective cohort; follow-up: 11.0 y (range 10.0–12.0)	86 pts aged < 61 y, who had undergone primary uncemented THA (53 M [62%], 33 F [38%] Age: 63 (range 40–72) BMI: 27 (range 18–39) Co NR	Primary uncemented THA	NR
Johnsson and Persson [47] 1986; Sweden; level of evidence: III	Retrospective; follow-up: 2.0 y	118 pts aged < 60 y with OA (76 M [64%], 42 F [36%]) Age: 54 (range 36–59) BMI and Co NR	Primary cemented THA	NR
Karampinas et al. [35] 2017; Greece; level of evidence: IV	Retrospective; follow-up: 2 y	Pts aged < 65 y with OA BFH group: *n* = 16 (11 M [69%], 5 F [31%]) Age: range 52–70 BMI and Co NR SMF group: *n* = 18 (11 M [61%], 7 F [39%]) Age: range 52–70 BMI and Co NR	Uncemented THA (posterior approach): BFH, SMF	Partial weight bearing allowed on postop day 2 Progress to full weight bearing as tolerated Use of crutches for 4 wks
Kleim et al. [12] 2015; UK; level of evidence: IV	Cross-sectional; follow-up: 1.8 ± 0.9 y	52 pts aged < 60 y with OA (23 M [44%], 29 F [56%]) Age: 52 ± 7.9 BMI and Co NR	Primary THA (not otherwise specified)	NR
Krischak et al. [48] 2013; Germany; level of evidence: III	Retrospective cohort; follow-up: 2.0 y	736 pts aged 18–60 y with OA (483 M [66%], 253 F [34%]) Age: 50.4 ± 6.2 (range 24.0–60.0) BMI and Co NR	Primary THA (not otherwise specified)	NR
Lefevre et al. [36] 2013; France; level of evidence: IV	Retrospective; follow-up: 8.8 ± 7.1 y	27 Judokas aged > 60 y with at least a black belt Age: 63 ± 7.2 Sex, BMI and Co NR	Primary THA (not otherwise specified)	NR
Leichtenberg et al. [13] 2016; the Netherlands; level of evidence: II	Prospective observational; follow-up: 1.0 y	67 pts aged < 65 y with OA + working preop (34 M [51%], 33 F [49%]) Age: 56 ± 6.6 BMI: 28 ± 6.0 Co NR	Primary THA (not otherwise specified)	NR
Mikkelsen et al. [49] 2014; Denmark; level of evidence: I	Non-randomized controlled; follow-up: 0.1 y	365 pts with OA undergoing THA (191 M [52%], 174 F [48%]) Age: 68.7 ± 10.0 BMI: 26.8 ± 4.5 Co NR	Primary THA (posterior approach)	Physiotherapy 1–2 times daily Home-based training program for 3 wks RG: traditional rehabilitation including movement restrictions UG: no movement restrictions
Mobasheri et al. [50] 2006; UK; level of evidence: III	Retrospective; follow-up: 3.0 y (range 0.5–10.0)	86 pts aged < 60 y with OA (56 M [65%], 30 F [35%]) Age: 51.4 (range 29.0–60.0) BMI and Co NR	Primary THA (not otherwise specified)	NR
Mont et al. [39] 1999; USA; level of evidence: III	Retrospective; follow-up: 8.0 y (range 2.0–22.0)	58 pts who were playing tennis and had undergone THA (50 M [86%], 8 F [14%]) Age: 62 (range 47–77) BMI and Co NR	Primary THA Fixation (*n*): Cemented 22 (29%), uncemented 48 (64%), hybrid 5 (7%)	NR
Nevitt et al. [62] 1984; USA; level of evidence: III	Retrospective; follow-up: 4.0 y	178 pts aged ≤ 60 y with degenerative, congenital or post-traumatic hip disorder (78 M [44%], 100 F [56%]) Age: 50 (range 21–60) BMI and Co NR	Primary THA (not otherwise specified)	NR
Pagnano et al. [51] 2006; USA; level of evidence: II	Retrospective; follow-up: minimum 0.5 y after second THA	26 pts with bilateral OA (10 M [38%], 16 F [62%]) Age: 69 (range 42–80) BMI and Co NR	Staged bilateral uncemented THA	Full weight-bearing as tolerated
Peak et al. [52] 2005; USA; level of evidence: I	Randomized prospective; follow-up: 0.5 y	265 pts undergoing primary THA (139 M [52%], 126 F [48%]) Age: 58.3 (range 14.0–88.0) BMI: RG, 29.3 (range 15.9–50.2); UG, 28.7 (range 17.6–45.7) Co NR	Uncemented THA (anterolateral approach)	Limited ROM (< 90° flexion) Full weight bearing as tolerated RG: traditional rehabilitation including full hip precautions 6 wks postop UG: no hip precautions
Poehling-Monaghan et al. [53] 2015; USA; level of evidence: III	Retrospective cohort; follow-up: 0.7 y	Pts with OA who had not undergone previous surgery DA THA: *n* = 126 (59 M [46%], 67 F [53%]) Age: 64.8 ± 12.4 BMI: 30.0 ± 5.5 Co NR MP THA: *n* = 96 (52 M [54%], 44 F [45%]) Age: 63.9 ± 12.5 BMI: 30.5 ± 6.0 Co NR	Primary THA: DA, MP	Rapid rehabilitation protocol Seen by a physical therapist twice daily Weight bearing allowed as tolerated with gait aids
Pons [54] 2010; Spain; level of evidence: III	Retrospective cohort; follow-up: 3.2 y (range 0.1–8.2)	128 pts with OA (90 M [70%], 38 F [30%]) Age: 57 (range 22–76) BMI and Co NR	Primary uncemented collum femoris preserving THA (posterolateral approach)	Immediate partial weight bearing Full weight bearing 3 wks postop
Pop et al. [61] 2016; Poland; level of evidence: IV	Retrospective; follow-up: 10.0 y	32 pts aged < 65 y at follow-up who underwent THA between 2003 and 2005 and did not experience postop complications (18 M [56%], 14 F [44%]) Age: 58 (range 39–65) BMI NR (38% overweight, 28% obese) Co NR	Uncemented THA (84%)	Postop rehabilitation: yes, 22; no, 10
Raguet et al. [41] 2015; France; level of evidence: III	Retrospective; follow-up: 8.0 y (range 1.0–19.0)	7 pts who underwent THA and practice ultrarunning (6 M [86%], 1 F [14%]) Age: 69.9 (range 61.0–86.0) BMI: 22.4 (range 19.0–25.0) Co NR	Uncemented THA (postero-lateral approach)	NR
Sankar et al. [55] 2013; Canada; level of evidence: II	Prospective cohort; follow-up: 1.0 y	190 pts aged 18–85 y with OA, who were working at baseline (100 M [53%], 90 F [47%]) Age: 56.1 ± 9.9 BMI: < 30: 120 (63%); ≥ 30: 69 (36%) Co NR	Primary THA (not otherwise specified)	NR
Schmidutz et al. [37] 2012; Germany; level of evidence: IV	Retrospective case study; follow-up: 2.7 y (range 2.0–4.2)	68 pts aged < 65 y, undergoing THA, head-neck-shaft angle > 120° (41 M [60%], 27 F [40%] Age: 55 (range 20–73) BMI: 26 (range 18–39) Co NR	Short stem hip arthroplasty (ceramic head)	Restrictions concerning weight bearing and ROM for the first 6 wks RTS recommendations based on consensus guidelines
Suarez et al. [56] 1996; Spain; level of evidence: IV	Retrospective case study; follow-up: unknown	747 pts aged 18–64 y who were working preop (598 M [80%], 149 F [20%]) Age: 46.9 (range 18.0–64.0) BMI and Co NR	Primary THA (not otherwise specified)	Rehabilitation was adapted to pt and could include: Kinesiotherapy postop day 1–10: hydrotherapy, ergo therapy, physical therapy for 4–5 wks
Suckel and Best [38] 2006; Germany; level of evidence: III	Retrospective; follow-up: 4.9 y (range 1.0–18.8)	16 pts (22 THAs) with OA who were playing golf preop Age: 65.7 (range 58.0–78.0) Sex, BMI, and Co NR	Primary THA (18 uncemented, 3 hybrid, 1 cemented)	NR
Tilbury et al. [57] 2015; the Netherlands; level of evidence: II	Prospective cohort; follow-up: 1.0 y	71 pts aged < 65 y with OA who had undergone THA + were working at baseline (37 M [52%], 34 F [48%]) Age: 56.0 ± 6.6 BMI: 27.8 ± 6.0 Co NR	Primary THA (not otherwise specified)	NR
Truszcynska et al. [58] 2013; Poland; level of evidence: III	Retrospective cohort; follow-up: 2.0 ± 1.5 y	54 pts aged < 65 y with OA who were working preop (29 M [54%], 25 F [46%]) Age: 55.9 ± 7.4 BMI and Co NR	Primary THA (not otherwise specified)	NR
Visuri et al. [59] 1987; Finland; level of evidence: III	Retrospective; follow-up: 4.2 y	539 pts who had undergone THA (166 M [31%], 373 F [69%]) Age range 25–84 BMI and Co NR	Primary THA (not otherwise specified)	NR
White [60] 1987; England; level of evidence: III	Retrospective; follow-up: 7.5 y (range 5.0–10.0)	33 pts aged < 45 y (12 M [36%], 21 F [64%]) Age: 38 (range 24–44) BMI and Co NR	Cemented THA (posterior approach)	NR

Study	Outcome measures	Preop (pre) activity + definition of preop	Postop activity	RTS + time to RTS	RTW + time to RTW	Confounding factors
Abe et al. [29] 2014; Japan; level of evidence: III	Sports participation (*n*)			48% Time to RTS: unknown	Unknown	Adjusted for in analysis: age, sex, BMI, operation type, bearing type, femoral head size, preop jogging Mentioned, not adjusted for: short term follow-up, motivation/reasons for not jogging
	Jogging	27	13			
	UCLA score	–	10			
		Definition: pre-surgery				
Arbuthnot et al. [30] 2007; UK; level of evidence: III	Sports participation (*n*)			86% Time to RTS: Return to practice: 4.1 mo (range 0–48) Return to play: 5.4 mo (range 0–60)	Unknown	Adjusted for in analysis: none Mentioned, not adjusted for: reasons for no return to golf, recall bias, surgeons’ advice
	Golf	66	57			
		Definition: pre-symptomatic and pre-surgery				
Atkinson et al. [42] 2010; UK; level of evidence: I	–	– Definition: pre-surgery	–	Unknown	Unknown	Adjusted for in analysis: age, ASA grade, bilateral THA (staged vs. single-episode) Mentioned, not adjusted for: none
				Time to RTS: Group 1: 24.8 wks; Group 2: 30.8 wks	Time to RTW: Part-time: Group 1: 13.8 wks; Group 2: 19.3 wks	
					Full-time: Group 1: 22.0 wks; Group 2: 35.8 wks	
Berger et al. [43]; 2004; USA; level of evidence: II	–	– Definition: pre-surgery	–	Unknown	100% Time to RTW: 8 days (range 1–20)	Adjusted for in analysis: none Mentioned, not adjusted for: selection bias, rapid recovery protocol, approach, surgeon’s advice
Bohm [44] 2010; Canada; level of evidence: II	–	– Definition: pre-surgery	–	Unknown	86% 20% of pts who were not working pre-op resumed work 2% stopped working Time to RTW: unknown	Adjusted for in analysis: sex, age, collecting disability insurance, preop job satisfaction, workload, Oxford Hip Score, functional limitations, education, personal income, self-employed, job motivation Mentioned, not adjusted for: none
Chatterji et al. [31] 2004; Australia; level of evidence: III	Sports participation (*n*):			93%^b^ RTS [% (wks until RTS)]	Unknown	Adjusted for in analysis: age, sex, preop sports participation Mentioned, not adjusted for: recall bias, physiotherapist’s advice, surgeon’s advice
	Overall	188	196	>100		
	Ice skating	0	0	–		
	Water skiing	0	0	–		
	Volleyball	0	0	–		
	Sailing	1	1	100 (?)		
	Horse riding	1	1	100 (14)		
	Croquet	2	0	0 (?)		
	Rowing	2	0	0 (12)		
	Snow skiing	4	1	25 (?)		
	Hiking	6	5	83 (58)		
	Jogging	7	1	14 (?)		
	Gardening	9	4	44 (9)		
	Aqua aerobics	17	32	>100 (9)		
	Bush walking	20	20	100 (21)		
	Tennis	14	1	7 (91)		
	Fishing	23	17	74 (20)		
	Cycling	28	18	64 (28)		
	Exercise classes	28	21	75 (16)		
	Bowling	36	28	78 (21)		
	Golf	39	26	67 (22)		
	Swimming	52	37	71 (8)		
	Exercise walking	145	169	>100 (10)		
	Grimby scale	3.5 ± 1.2 Definition: pre-surgery	Unknown			
Clyde et al. [45] 2013; USA; level of evidence: III	Occupational category (*n*):			Unknown	76% Same job 91%; change of job 9% Time to RTW primary THA: 17.3 wks (range 2.0–156.0)	Adjusted for in analysis: sex, age, BMI, follow-up period, workload Mentioned, not adjusted for: recall bias, socioeconomic status, postop complications
	Unemployed	1	11			
	Non-manual labor (little physical activity)	14	13			
	Moderate labor (lifting < 20 lb)	17	14			
	Strenuous labor (lifting 20–50 lb)	11 Definition of preop: pre-surgery	5			
Danielsson [46] 1965; Sweden; level of evidence: II	–	– Definition: pre-surgery	–	Unknown	57% Previous work: 18%; light manual work: 39% Time to RTW: unknown	Adjusted for in analysis: workload Mentioned, not adjusted for: none
Del Piccolo et al. [40] 2016; Italy; level of evidence: III	Intense sports activities (including skiing, free running, tennis and contact sports, *n*):			Similar RTS for light and medium level activities RTS (%)	Unknown	Adjusted for in analysis: age, BMI, follow-up, THA design Mentioned, not adjusted for: not randomized, general applicability, short follow-up
	Conventional stem	19	8	40%		
	Short stem	9	4	44%		
		Definition: pre-symptomatic		Time to RTS: unknown		
Dubs et al. [28] 1983; Switzerland; level of evidence: III	Sports participation (*n*):			RTS (%)	RTW (%) 93 29 80 > 100 – Time to RTW: unknown	Adjusted for in analysis: implant loosening Mentioned, not adjusted for: sex (only M), workload, surgeon’s advice
	Overall	86	61	71		
	Hiking/climbing	44	41	93		
	Skiing	43	4	9		
	Swimming	28	35	> 100		
	Running	17	17	100		
	Ball sport	16	0	0		
	Cycling	14	10	25		
	Tennis	9	4	44		
	Riding	9	1	11		
	Light athletics	6	0	0		
	Wrestling	2	0	0		
	Rowing	2	0	0		
	Sailing	1	3	> 100		
	Boxing	1	0	0		
	Canoeing	1	1	100		
	Workload (*n*)			Time to RTS: unknown		
	Overall	108	100			
	Strenuous	24	7			
	Medium	41	33			
	Light	43	60			
	Retired	2	10			
		Definition: pre-surgery				
Hara et al. [32] 2017; Japan; level of evidence: III	Sports participation (*n*):			> 100 RTS (%)	Unknown	Adjusted for in analysis: age, BMI, sex, preop sports participation, preop UCLA score Mentioned, not adjusted for: none
	Overall	288	318	> 100		
	Walking	48	84	> 100		
	Swimming	50	61	> 100		
	Gymnastics	23	50	> 100		
	Strength/muscle training	14	26	> 100		
	Cycling	23	21	91		
	Golf	23	13	57		
	Dancing	12	6	50		
	Bowling	10	4	40		
	Racket games	17	6	35		
	Aerobics	6	2	33		
	Jogging	10	7	70		
	Ball games	24	7	29		
	Other	28	32	> 100		
	UCLA score			Time to RTS: unknown		
	RTS, yes	4.3 ± 2.3	5.7 ± 1.8			
	RTS, no	3.5 ± 2.0	4.1 ± 1.5 (*p* < 0.001)			
		Definition: pre-surgery				
Huch et al. [33] 2005; Germany; level of evidence: II	Sports participation (*n*):	Lifetime/preop		> 100 Lifetime/preop	Unknown	Adjusted for in analysis: sex, age, smoking, workload, reasons for no-RTS Mentioned, not adjusted for: BMI, surgeon’s advice, comorbidities, comparability responders and non-responders
	Overall	408/151	218	53/> 100		
	Biking	248/42	181	73/> 100		
	Hiking	227/4	161	71/> 100		
	Swimming	193/38	151	78/> 100		
	Downhill skiing	126/0	17	13/> 100		
	Gymnastics	88/21	57	65/> 100		
	Cross country skiing, jogging	71/0	20	28/> 100		
	Tennis	46/4	10	22/> 100		
	Dancing	25/0	20	80/> 100		
		Definition: lifetime and pre-surgery				
				Time to RTS: unknown		
Innmann et al. [34] 2016; Germany; level of evidence: III	Sports participation (*n*):			RTS:	Unknown	Adjusted for in analysis: sex, age Mentioned, not adjusted for: recall bias, selection bias, lack of preop data for SF-36 questionnaire
	Overall	86	77	89		
	Cycling	25	36	> 100		
	Hiking	17	12	71		
	Exercise walking	14	16	> 100		
	Tennis	15	5	33		
	Soccer	10	0	–		
	Fitness/weight lifting	8	14	> 100		
	Jogging	7	4	57		
	Downhill skiing	7	1	14		
	Gymnastics	6	15	> 100		
	Cross-country skiing	6	1	17		
	Swimming	5	15	> 100		
	Basketball/handball	5	0	–		
	Table tennis	4	1	25		
	Mountain climbing	4	1	25		
	Nordic walking	2	6	> 100		
	Aqua-aerobics	2	5	> 100		
	Dancing	2	3	> 100		
	No sports	20	18	90		
	UCLA (range)	3.8 ± 1.6 (1–9) Definition: pre-symptomatic	6.2 ± 1.5 (3–10) (*p* < 0.001)	Time to RTS: < 4 wks: 22%; 1–3 mo: 29%; 3–6 mo: 22%; > 6 mo: 6%		
Johnsson and Persson [47] 1986; Sweden; level of evidence: III	Type of work (*n*):			Unknown	66% Time to RTW: within 2 y	Adjusted for in analysis: sex, age, sick leave, workload, bilateral THA, reason for no RTW Mentioned, not adjusted for: rehabilitation
	Household	2	3			
	Light work	11	19			
	Moderate work	25	32			
	Heavy work	31	15			
		Definition: 2 y preop				
Karampinas et al. [35] 2017; Greece; level of evidence: IV	Sports participation (*n*):			RTS:	Unknown	Adjusted for in analysis: none Mentioned, not adjusted for: type of prosthesis, pt fear of complications, surgeon’s advice
	BFH group					
	Low impact	8	8	100%		
	Intermediate impact	6	8	> 100%		
	High impact	2	–	0%		
	SMF group					
	Low-impact	8	10	> 100%		
	Intermediate impact	7	5	71%		
	High impact	3	3	100%		
	UCLA score			Time to RTS: 16.5 wks (range 12.0–26.0)		
	BFH group	3.5	6.7			
	SMF group	3.8	7.9 (*p* = NR)			
	HHS score					
	BFH group	56.5	93.7			
	SMF group	48.5	94.4 (*p* = NS)			
		Definition: pre-surgery				
Kleim et al. [12] 2015; UK; level of evidence: IV	Manual requirements (*n*):			Unknown	75% Time to RTW: 12 ± 5 wks Preop sick leave: 15 ± 4 wks No preop sick leave: 10 ± 1 wks	Adjusted for in analysis: workload, preop sick leave, education Mentioned, not adjusted for: time to RTW, selection bias, general applicability, motivation
	0 = no manual requirements	24	–			
	1 = some manual requirement	17	–			
	2 = heavy manual work	4	–			
		Definition: pre-surgery				
Krischak et al. [48] 2013; Germany; level of evidence: III	**–**	**–** Definition: pre-surgery	**–**	Unknown	85% 83% same job 17% different job Time to RTW: unknown	Adjusted for in analysis: workload, age Mentioned, not adjusted for: THA approach
Lefevre et al. [36] 2013; France; level of evidence: IV	**–**	**–** Definition: unknown	–	82% Time to RTS: 3.9 mo ± 2.7 All pts stopped participating at a competitive level	Unknown	Adjusted for in analysis: age Mentioned, not adjusted for: selection bias, motivation, experience, surgeon’s advice
Leichtenberg et al. [13] 2016; the Netherlands; level of evidence: II	Type of work (*n*):			Unknown	Full RTW: 79%; partial RTW: 13%; no RTW: 7%^d^; time to RTW: unknown	Adjusted for in analysis: type of prosthesis, sex, age, education, self-employed, preop h worked, workload, preop work adaptions, preop workers compensation, preop sick leave Mentioned, not adjusted for: recall bias, baseline HOOS
	Light	41	33			
	Medium	14	10			
	Heavy	4 Definition: presurgery	3 (*p* = 0.672)			
Mikkelsen et al. [49] 2014; Denmark; level of evidence: I	Pts working (*n*)			Unknown	45% Time to RTW: unknown	Adjusted for in analysis: rehabilitation protocol Mentioned, not adjusted for: selection bias, short inclusion and follow-up period, lack of blinding, information bias, missing data
	RG	37	12			
	UG	54	29 (*p* = 0.045)			
		Definition: pre-surgery				
Mobasheri et al. [50] 2006; UK; level of evidence: III	–	– Definition: pre-surgery	–	Unknown	RTW: employed preop: 96%; unemployed preop: 43% Time to RTW: employed preop: 10.5 wks; unemployed preop: 35.0 wks	Adjusted for in analysis: reasons for no RTW, self-employment, sex, > 1 y unemployed preop Mentioned, not adjusted for: preop sick-leave
Mont et al. [39] 1999; USA; level of evidence: III	Sports participation (*n*):			RTS (%)	Unknown	Adjusted for in analysis: level of play, singles vs doubles, return to competitive play, motivation Mentioned, not adjusted for: fixation, recall bias, selection bias, surgeon’s advice
	Tennis	58	58	100%		
		Definition: pre-surgery		Time to RTS: 6.7 mo (range 1.0–12.0)		
Nevitt et al. [62] 1984; USA; level of evidence: III	Work status (*n*):	Lifetime/pre-surgery	1 y postop/4 y postop	Unknown	RTW: 1 y postop: 68%; 4 y postop: 63% 35% limitations in physical activities; 41% limited in the kind or the amount of their work Time to RTW: unknown	Adjusted for in analysis: sex, age, diagnosis, prior hip surgery, y of THA, joint pain, uni- or bilateral THA, preop work status Mentioned, not adjusted for: timing of surgery, general unemployment rates, prosthesis type
	Working	139/81	95/87			
	Disabled	NR/58	44/52			
		68% limitations in physical activities 44% limited in the kind or the amount of their work Definition: lifetime and 1 mo pre-surgery				
Pagnano et al. [51] 2006; USA; level of evidence: II	–	– Definition: pre-surgery	–	Unknown	100% Time to RTW: mini-posterior THA: 38 days (range 14–90) Two-incision THA: 42 days (range 9–56)	Adjusted for in analysis: approach (mini-posterior vs. two-incision) Mentioned, not adjusted for: influence of the first THA, complications
Peak et al. [52] 2005; USA; level of evidence: I	Working (*n*):			Unknown	RTW: RG 95%; UG 100% (*p* = NS) Time to RTW (wks): RG 9.5; UG 6.5 (*p* < 0.001)	Adjusted for in analysis: rehabilitation protocol Mentioned, not adjusted for: selection bias, approach
	RG	85	81			
	UG	98	98			
		Definition: pre-surgery				
Poehling-Monaghan et al. [53] 2015; USA; level of evidence: III	–	– Definition: pre-surgery	–	Unknown	RTW at 8 weeks: DA 69%; MP: 97% (*p* < 0.01) Time to RTW: unknown	Adjusted for in analysis: THA approach Mentioned, not adjusted for: mismatch in experience
Pons [54] 2010; Spain; level of evidence: III	–	– Definition: pre-surgery	–	Unknown	96% Time to RTW: unknown	Adjusted for in analysis: none Mentioned, not adjusted for: none
Pop et al. [61] 2016; Poland; level of evidence: IV	Employment status 1 y preop 1 y postop 10 y postop	Unknown Definition: 1 y preop, 1 y postop, 10 y postop	Unknown	Unknown	Employment status: 1 y preop: yes 28 (88%), no 0 (0%) Disability/retirement: 4 (12%) 1 y postop: yes 15 (47%), no 4 (13%) Disability/retirement: 13 (41%) 10 y postop: yes 13 (41%), no 1 (3%) Disability/retirement: 18 (57%) Time to RTW: unknown	Adjusted for in analysis: none Mentioned, not adjusted for: functional capacity, BMI, sex, place of residence, level of physical capacity, comorbidities, supervised rehabilitation
Raguet et al. [41] 2015; France; level of evidence: III	Sports participation (*n*):			RTS (%)	Unknown	Adjusted for in analysis: none Mentioned, not adjusted for: age, surgeon’s advice, bearing type, postop pain, fear of luxation
	Ultrarunning	7	7	100		
	UCLA score	–	10	Time to RTS: unknown		
		Definition: pre-symptomatic				
Sankar et al. [55] 2013; Canada; level of evidence: II	Physical demands at work (*n*):			Unknown	87% Time to RTW: ≤ 1 mo: 39%; ≤ 3 mo: 36%; 6–12 mo: 25%	Adjusted for in analysis: age, sex, education level, job sector, physical demands Mentioned, not adjusted for: workplace accommodations
	Low demand	88	78			
	High demand	74	67			
	Unclassified	22	18			
		Definition: pre-surgery				
Schmidutz et al. [37] 2012; Germany; level of evidence: IV	Sport participation (%): Low impact			98% RTS (%)	Unknown	Adjusted for in analysis: sex, age, level of impact Mentioned, not adjusted for: recall bias, surgeons’ advice
	Cycling	69	69	100		
	Hiking	54	57	> 100		
	Nordic walking	12	18	> 100		
	Gymnastics	22	26	> 100		
	Fitness/weight training	22	38	> 100		
	Dancing	22	22	100		
	Swimming	57	56	98		
	Golf	1	1	100		
	Intermediate impact					
	Badminton	7	3	43		
	Inline skating	4	1	25		
	Tennis	15	3	20		
	Downhill skiing	24	16	67		
	Cross-country skiing	21	15	71		
	Riding	7	3	43		
	Martial arts	4	1	25		
	Bowling	10	6	60		
	Rock climbing	1	1	100		
	High impact					
	Jogging	9	3	33		
	Handball	1	0	–		
	Volleyball	7	3	43		
	Basketball	4	1	25		
	Soccer	9	1	11		
	Squash	7	0	-		
	UCLA score	Unknown Definition: pre-surgery	7.6 ± 1.9 (range 3–10)	Time to RTS: 1–2 mo: 27%; 3–4 mo: 25%; 5 to ≥ 6 mo: 48%		
Suarez et al. [56] 1996; Spain; level of evidence: IV	Type of work (*n*)			Unknown	25% RTW (%)	Adjusted for in analysis: sex, age, workload, education, social security type, environment (rural/urban), family structure, underlying illness Mentioned, not adjusted for: cultural background
	Heavy work	456	7		1.5	
	Moderate work	134	84		63	
	Light work	157	143		91	
		Definition: pre-surgery			Time to RTW: unknown	
Suckel and Best [38] 2006; Germany; level of evidence: III	Sports participation (*n*):			RTS (%)	Unknown	Adjusted for in analysis: none Mentioned, not adjusted for: side, surgeon’s advice, risk of wear, golf experience
	Golf	16	16	100		
		Definition: pre-surgery		Time to RTS: unknown		
Tilbury et al. [57] 2015; the Netherlands; level of evidence: II	–	– Definition: pre-surgery	–	Unknown	90% Time to RTW: 12.5 ± 7.6 wks Reason for no RTW: sick leave 2 (3%); retired 2 (3%); unknown 3 (4%)	Adjusted for in analysis: sex, age, BMI, education, radiographic severity, HOOS, EQ-5D score, Oxford Hip Score, SF-36 score Mentioned, not adjusted for: recall bias, postop complications
Truszcynska et al. [58] 2013; Poland; level of evidence: III	–	– Definition: pre-surgery	–	Unknown	59% All of these pts returned to their preop employment level Time to RTW: 94% within 6 mo	Adjusted for in analysis: sex, age, satisfaction with job, education level, compliance with exercise program, mental health Mentioned, not adjusted for: none
Visuri et al. [59] 1987; Finland; level of evidence: III	Type of work (*n*):			Unknown	67% Time to RTW: unknown	Adjusted for in analysis: age, sex, workload, social class, primary diagnosis, walking ability, pensioner groups Mentioned, not adjusted for: type of prosthesis
	Laborers	303	27			
	Service workers	69	22			
	Mental workers	99	18			
	Housewives	68	–			
		Definition: pre-surgery				
White [60] 1987; England; level of evidence: III	Workload (*n*) Heavy manual work	8 Definition: Lifetime	6	Unknown	92% 3 pts took up employment for the first time following THA Time to RTW: unknown	Adjusted for in analysis: diagnosis, workload Mentioned, not adjusted for: previous surgery, weight gain, patient activity

Levels of evidence: I = randomized controlled study, II = prospective study, III = retrospective (comparative) study, IV = retrospective case series

*ASA* American Society of Anesthesiologists, *BFH* big femoral head arthroplasties, *BMI* body mass index, *Co* co-morbidities, *DA* direct posterior, *EQ-5D* EuroQol-5D, *F* female, *HHS* Harris Hip Score, *HOOS* Hip disability and Osteoarthritis Outcome Score, *M* male, *mo* months, *MP* mini-posterior, *NR* not reported, *NS* not significant, *OA* osteoarthritis, *postop* postoperative, *preop* preoperative, *pts* patients, *RG* restricted group, *ROM* range of motion, *RTS* return to sports, *RTW* return to work, *SMF* short modular femoral hip system, *THA* total hip arthroplasty, *UCLA* University of California, Los Angeles, *UG* unrestricted group, *wk(s)* week(s), *y* years

^a^Age is presented in years unless otherwise indicated and BMI is presented in kg/m^2^

^b^Chatterji et al. reported return to sports of 175 of 188 patients (93%) performing sports preoperatively, plus 21 patients who took up at least one sport postoperatively

^c^Del Picolo et al. used different numbers in the abstract and in the methods section. We report number that was stated in the methods section

^d^Total RTW percentage is 99% in original article

The total number of included patients was 6485, with 3066 males (47%) and 3016 females (47%). Five studies did not report the sex distribution in their cohort, thus sex was unknown for 6% of the included patients [30, 31, 36, 38, 40]. The mean duration of follow-up was 3.8 years (range 0.25–11), with one study not reporting time to follow-up [56]. The mean age across studies ranged from 38 to 71 years, with a total age range of 14–98 years. Patients’ BMI was specified in 14 studies, with mean BMI varying from 22 to 32 kg/m^2^. One study provided information on comorbidities [33]. The authors described that, of the included 420 patients, 7% had diabetes, 45% had hypertension, and 11% had gout. The approach was described in ten studies: one study used a direct anterior approach, two used an anterolateral approach, three used a posterolateral approach, three used a posterior approach, and one used a two-incision approach. The rehabilitation protocol was described in 13 studies. Immediate full weight bearing was allowed in seven studies [29, 32, 35, 43, 51–53] and partial weight bearing was recommended for 3–6 weeks in four studies [37, 40, 46, 54]. Two studies compared restricted and unrestricted movement protocols [49, 52]. The use of crutches was advised for 4–8 weeks in three studies [32, 35, 40].

### Methodological Quality

Table 2 summarizes the results of the quality assessment. The methodological quality was rated as high in 11 studies, moderate in 16 studies, and low in ten studies. The lowest risk of bias was found for the item “selection of the cohort” (36 studies scored a star), the item “ascertainment of exposure” (34 studies scored a star), and the item “demonstration that the outcome of interest (RTS/RTW) was not present at the start of the study” (36 studies scored a star). The highest risk of bias was found for the item “assessment of outcome,” for which ten studies scored a star (Table 2).

**Table 2:** Methodological assessment according to the Newcastle–Ottawa scale

Study	Selection				Comparability	Outcome			Total score^a^
	Representativeness	Selection	Ascertainment	Outcome of interest		Assessment	FU	Adequacy of FU	
Abe et al. [29]	*	*	*	*	**	–	*	–	7
Arbuthnot et al. [30]	*	*	*	*	*	–	*	*	7
Atkinson et al. [42]	*	*	*	*	*	–	*	*	7
Berger et al. [43]	*	*	*	*	–	*	–	*	6
Bohm [44]	*	*	*	*	**	–	*	*	8
Chatterji et al. [31]	*	*	*	*	**	–	*	–	7
Clyde et al. [45]	–	*	*	*	**	–	*	–	6
Danielsson [46]	*	*	*	*	*	–	*	*	7
Del Piccolo et al. [40]	*	*	*	*	*	*	*	–	7
Dubs et al. [28]	–	*	*	*	–	–	*	*	5
Hara et al. [32]	*	*	*	*	**	–	*	–	7
Huch et al. [33]	*	*	*	*	**	–	*	*	8
Innmann et al. [34]	*	*	*	*	*	–	*	*	7
Johnsson and Persson [47]	*	*	*	*	**	*	*	*	9
Karampinas et al. [35]	–	*	*	*	*	–	*	–	5
Kleim et al. [12]	*	*	*	*	**	–	–	–	6
Kirschak et al. [48]	*	*	*	*	**	*	*	*	9
Lefevre et al. [36]	–	*	–	*	*	–	*	–	4
Leichtenberg et al. [13]	*	*	*	*	**	–	*	*	8
Mikkelsen et al. [49]	–	*	*	*	*	–	–	*	5
Mobasheri et al. [50]	*	*	*	*	**	–	*	*	8
Mont et al. [39]	–	*	–	*	*	–	*	–	4
Nevitt et al. [62]	*	*	*	*	**	–	*	*	8
Pagnano et al. [51]	–	*	*	*	*	–	–	*	5
Peak et al. [52]	*	*	*	*	–	*	–	–	5
Poehling-Monaghan et al. [53]	*	–	*	–	–	–	–	–	2
Pons [54]	*	*	*	*	–	*	*	–	6
Pop et al. [61]	–	*	*	*	*	–	*	–	5
Raguet et al. [41]	–	*	*	*	*	–	*	*	6
Sankar et al. [55]	*	*	*	*	**	–	*	*	8
Schmidutz et al. [37]	*	*	*	*	**	–	*	*	8
Suarez et al. [56]	*	*	*	*	**	*	–	–	7
Suckel et al. [38]	–	*	–	*	–	–	*	–	3
Tilbury et al. [57]	*	*	*	*	**	–	*	*	8
Truszczynska et al. [58]	*	*	*	*	*	*	*	–	7
Visuri et al. [59]	*	*	*	*	**	*	*	*	9
White [60]	–	*	*	*	*	*	*	*	7

*FU* follow-up

– indicates no stars

^a^We considered a study to be of high quality when the total score was eight or nine stars, moderate quality when the total score was six or seven stars, and low quality when the total score was five stars or fewer

### Return to Sports

Of 15 studies that reported RTS and time to RTS, 14 reported the percentage of patients that RTS. Mean RTS percentages varied from 43 to > 144%, the latter indicating that more patients participated in sports activities postoperatively than preoperatively (Table 1). Ten studies describing the preoperative sports level as the moment before surgery (pre-surgery level) reported RTS percentages from 48 to > 100%. Four studies describing preoperative sports participation as the moment before the onset of restricting hip symptoms (pre-symptomatic level) reported that 43, 82, 86, and 100% could RTS [30, 34, 40, 41]. For the two high-quality studies, Huch et al. [33] and Schmidutz et al. [37], RTS was > 100% and 98%, respectively, relative to the pre-surgery level. In addition, Huch et al. [33] reported RTS with lifetime sports participation as a reference level and found an average RTS of 53%. Five studies reported time to RTS, with the average being 21.0 weeks (range 15.5–28.0) [30, 35, 36, 39, 42]. No studies with a low risk of bias reported time to RTS. In addition, Chatterji et al. [31], Innmann et al. [34], and Schmidutz et al. [37] reported the cumulative percentage of patients that had returned at different time points (Table 1).

Data could be pooled for 14 studies that reported exact numbers of patients participating in sports pre- and postoperatively (Table 3), including 2318 patients (60% male, median age 62.9 years). Overall, RTS was 100%, but this percentage depended on the definition of the preoperative sports level. Average return to pre-surgery sports level was 104%, and average return to pre-symptomatic sports level was 82%. Both high-quality studies reported the return to pre-surgery sports level and found an average RTS of 131% (Table 3). In addition, one high-quality study also reported return to lifetime sports level, with an average RTS of 53% (Table 3).

**Table 3:** Pooled data for number of patients participating in any sport pre- and postoperatively

Preoperative reference for RTS	No. of pts participating in any sport		RTS (%)
	Preoperatively	Postoperatively	
Overall (14 studies)	1125	1130	100
Pre-surgery participation as reference for RTS (10 studies)	938	977	104
Pre-symptomatic participation as reference for RTS (4 studies)	187	153	82
High-quality studies; pre-surgery participation as reference for RTS (2 studies)	214	280	131
High-quality studies; lifetime participation as reference for RTS^a^ (1 study)	408	218	53

*No.* number, *pt(s)* patient(s), *RTS* return to sports

^a^Huch et al. [33] reported both the pre-surgery and lifetime sports participation

In total, 11 studies described specific numbers of sports that were practiced pre- and postoperatively (Table 4), including 1605 patients (65% male, median age 63.0 years). Preoperatively, 1605 patients practiced an average of 1.1 sports, including 62% low-impact sports, 24% intermediate-impact sports, and 14% high-impact sports. Postoperatively, 1605 patients practiced an average of 1.0 sports, including 69% low-impact sports, 23% intermediate-impact sports, and 8% high-impact sports (Table 4). In the high-quality studies, 488 patients practiced an average of 1.3 sports preoperatively, including 52% low-impact sports, 37% intermediate-impact sports, and 11% high-impact sports. Postoperatively, 488 patients practiced an average of 1.1 sports, including 57% low-impact sports, 40% intermediate-impact sports, and 3% high-impact sports.

**Table 4:** Pooled data for pre- and postoperative sports participation for different types of sport impact levels

Impact level	Preoperative sports participation (11 studies)			Postoperative sports participation (11 studies)		
	Sports (*n*)	Pts (*n*)	Average sports/pt, *n* (%)	Sports (*n*)	Pts (*n*)	Average sports/pt, *n* (%)
Low (e.g., cycling, swimming, golfing)	1115	1605	0.69 (62)	1090	1605	0.68 (69)
Intermediate (e.g., hiking, downhill skiing)	427	1605	0.27 (24)	372	1605	0.23 (23)
High (e.g., tennis, running, ball sports)	250	1605	0.16 (14)	122	1605	0.08 (8)
Total	1792	1605	1.12	1584	1605	0.99

*pt(s)* patient(s)

### Return to Work

Of 24 studies that reported RTW and time to RTW, 23 reported the mean percentage of RTW, which varied from 25 to > 122%, the latter indicating that more patients worked postoperatively than preoperatively (Table 1). For the high-quality studies, mean RTW percentages were 66, 67, 68, 85, 87, 87, 90, 93, and 122%, respectively [13, 44, 47, 48, 50, 55, 57, 59, 62]. Time to RTW was reported in eight studies, and the mean duration of inability to work varied from 1 to 17 weeks. Pooling of studies, including 3536 patients (53% male, median age 52.7 years), showed that 3097 patients worked preoperatively and overall RTW was 69% (Table 5). For the high-quality studies, a mean RTW of 83% was found. A large difference in RTW was found between studies published before or after 2000, with a mean RTW of 44% for studies published in or before 2000 and 86% for those published after 2000 (Table 5). Pooling of studies that reported time to RTW showed a mean inability to work of 8.9 weeks. For the high-quality studies (*n* = 2), a mean inability to work of 11.4 weeks was found [50, 57].

**Table 5:** Pooled data for return to work and average duration of inability to work

	Number of working patients				Time to RTW	
	Preoperative (*n*)	Postoperative (*n*)	RTW (%)		Patients (*n*)	Inability to work (weeks)
Overall (23 studies)	3097	2138	69	Overall (8 studies)	746	8.9
High quality (9 studies)	1492	1242	83	High quality (2 studies)	157	11.4
Published in or before 2000 (16 studies)	1238	548	44	Published in or before 2000 (0 studies)	–	–
Published after 2000 (7 studies)	1859	1590	86	Published after 2000 (8 studies)	746	8.9

*RTW* return to work

### Secondary Outcome Measures of Physical Activity

The UCLA activity score was reported in six studies. Abe et al. [29] compared postoperative joggers with non-joggers and found a significantly higher postoperative UCLA score (mean ± standard deviation [SD]) of 10 ± 0 for joggers (*n* = 23) compared with 6.6 ± 2.4 for non-joggers (*n* = 585). Hara et al. [32] found that both preoperative and postoperative UCLA scores were significantly higher in patients who participated in sports postoperatively than in those who did not: 4.3 ± 2.3 versus 3.5 ± 2.0 preoperatively and 5.7 ± 1.8 versus 4.1 ± 1.5 postoperatively. Innmann et al. [34] found a significant increase in UCLA score 11 years after THA, from 3.8 ± 1.6 preoperatively to 6.2 ± 1.5 postoperatively. Karampinas et al. [35] reported that UCLA scores significantly improved from 3.5 to 6.7 (SD unknown) in the big femoral head group and from 3.8 to 7.9 (SD unknown) in the short metaphyseal stem group. Raguet et al. [41] found a postoperative UCLA score of 10 for seven patients participating in ultrarunning. Lastly, Schmidutz et al. [37] found a postoperative UCLA score of 7.6 ± 1.9 after a mean follow-up of 2.7 years. The Grimby scale was reported in one study. Chatterji et al. [31] found a mean postoperative Grimby scale score of 3.5 ± 1.2, at 1–2 years after surgery.

### Confounding Factors: Return to Sports

Of 15 studies reporting RTS, ten analyzed the effect of one or more confounding factors on RTS. Age was analyzed in eight studies, of which three found an age-dependent decline in RTS [29, 31, 33] and five found no association [32, 34, 37, 40, 42]. Sex was analyzed in five studies: two found a higher RTS in men than in women [29, 33], and three found no association [31, 34, 37]. BMI was analyzed in three studies, which found no association with RTS [29, 32, 40]. Four studies analyzed preoperative sports participation, and all found higher RTS in patients who had already participated in sports preoperatively [29, 31–33]. Additionally, Hara et al. [32] found that a higher preoperative UCLA score was associated with higher RTS. Lastly, one study analyzed the level of impact and found the chance of returning to intermediate- and high-impact sports activities was lower than that of returning to low-impact activities [37].

### Confounding Factors: Return to Work

Of 24 studies reporting RTW, 22 analyzed the effect of one or more confounding factors on RTW. Age was analyzed in 13 studies, of which four found higher RTW in younger patients [13, 44, 48, 59] and nine found no association [36, 42, 45, 47, 55–58, 62]. Sex was analyzed in 12 studies, of which two found that men returned to work faster than did women [50, 55] and ten found no association [13, 44, 45, 47, 56–59, 61, 62]. The three studies that analyzed BMI found no association with RTW [45, 57, 61]. Workload was analyzed in 11 studies, of which six found that higher preoperative workload resulted in lower RTW [45, 47, 48, 56, 59, 60] and two found that higher workload resulted in longer postoperative inability to work [12, 55]. Three studies found no association between workload and RTW [13, 44, 46]. Additionally, three studies found that patients with higher education returned to work faster and more often [12, 13, 55], and one study found that patients with elementary school education returned to work considerably less often [56]. Three studies found no association between education level and RTW [44, 57, 58]. Five studies analyzed preoperative sick leave and found it to be associated with lower RTW [12, 13, 47, 50, 62]. Additionally, one study found that patients who collected a disability insurance preoperatively were less likely to RTW [44]. Self-employment was analyzed in three studies, of which one found that self-employment resulted in partial or no RTW [13] and two studies found no association [44, 50]. Job satisfaction was analyzed in two studies, one of which found that 97% of patients who returned to work were satisfied with their job [58]. In contrast, one study found lower job satisfaction in patients who did RTW [44]. THA approach was analyzed in three studies, one of which found that the mini-posterior approach resulted in higher RTW than did the direct anterior approach [53]. Two other studies found no association [44, 51]. Two studies investigating a restricted compared with an unrestricted rehabilitation protocol found that patients in the unrestricted rehabilitation protocol returned to work more often [49] and faster [52] than those with a restricted protocol.

## Discussion

The aim of the present systematic review and meta-analysis was to investigate the extent of RTS and RTW after THA. Our most important finding was that a large percentage of patients returned to sports and work after THA. Concerning sports participation, the percentage of patients returning to any type of sports activity varied from 43 to > 100%, indicating that more patients participated in sports postoperatively than preoperatively. The average time to RTS was 21 weeks. We also found a decrease in participation in high-impact sports activities and a corresponding increase in participation in low-impact activities. Concerning work resumption, the percentage of patients who could RTW varied from 25 to > 100%, and the average time to RTW was 9 weeks.

### Return to Sports

Our meta-analysis revealed an overall RTS percentage of 100%. However, this percentage varied considerably with the preoperative reference point that was used to define sports participation. For instance, 104% of patients returned to their pre-surgery sports level, whereas only 82% returned to their pre-symptomatic level. As stated, we believe that the pre-symptomatic level represents a more appropriate reference point for RTS, since many patients limit their sports participation before joint replacement because of pain and functional limitations [24, 25]. Still, our finding that more patients participated in sports postoperatively compared with their pre-surgery sports level, with an RTS of 104%, is encouraging. Both high-quality studies used the pre-surgery level and not the pre-symptomatic level as a reference and consequently found very high RTS percentages (98 and 144%) [33, 37]. Huch et al. [33] also compared lifetime sports participation with postoperative sports participation and found an overall RTS of 53%. However, this is likely an underestimation, since lifetime participation also includes sports that patients practiced in their youth and had stopped practicing for reasons unrelated to their hip. Overall, an RTS of 82% after THA seems the best estimate, which is in accordance with findings in patients undergoing knee arthroplasty and knee osteotomy [24, 25].

### Return to Work

Regarding return to work, our meta-analysis revealed an overall RTW of 69%. The previous systematic review by Tilbury et al. [11] did not pool RTW data, hampering the comparison with the present review. However, the authors described that RTW ranged from 25 to 95% in seven studies. For the present review, we found 23 studies reporting RTW percentages, which varied from 25 to > 100%. Thus, the newly published studies that we identified reported a range similar to that in the previous review [11]. Given the increasing number of THAs performed in working age patients in the last 2 decades [27], we compared RTW percentages for studies published before and after 2000. As expected, the pooled RTW percentage was considerably higher in studies published after 2000 (86 vs. 44%). Apart from the increased utilization of THA in patients of working age, this difference might also be attributed to the large increase in less physically demanding jobs in developed countries as well as more liberal recommendations concerning RTW after THA. This was illustrated by findings of two studies describing differences in RTW and time to RTW between a restricted and unrestricted rehabilitation protocol [49, 52]. Mikkelsen et al. [49] found higher RTW in an unrestricted group (no movement restrictions, RTW of 54%) than in a restricted group (restricted hip movement for 3 months; RTW of 32%). Peak et al. [52] found that patients in an unrestricted group (no movement restrictions) returned to work after 6 weeks compared with 9 weeks in a restricted group (movement restrictions for 6 weeks). Interestingly, none of the other included studies mentioned the effect of rehabilitation protocol or surgeons’ advice concerning RTW. Thus, the effect of different rehabilitation protocols and surgeons’ recommendations on RTW and time to RTW is an important topic for future studies. Lastly, one included study reported that 5 of 67 patients (7%) did not RTW, whereas nine patients (13%) could only partially RTW [13]. Since most patients expressed a preoperative desire to fully RTW, this subtotal loss of work capacity might be clinically relevant. Therefore, future studies should aim to distinguish between a full and partial RTW after hip arthroplasty.

### Prognostic Factors

#### Prognostic Factors for Return to Sports

Regarding RTS, increasing age was associated with a decline in RTS in three studies, whereas five studies found no association. However, of those five studies, three limited their inclusion to patients aged < 60 years [34, 37, 40] and one study included a small number of patients [42], thus limiting statistical validity. In general, above the age of 65 years, a postoperative decline in sports participation might be expected for THA patients. An important prognostic factor for RTS is preoperative sports participation, which was analyzed by five studies, all of which found higher RTS in patients who had participated in sports preoperatively. This is in line with findings in patients undergoing knee arthroplasty and knee osteotomy [24, 25]. Only one study analyzed the impact level of a sport; the authors found that participation in intermediate- and high-impact sports was associated with lower RTS than participation in low-impact sports.

Remarkably, patients’ motivation to RTS, which is a proven prognostic factor for RTS in patients undergoing hip and knee arthroplasty [24, 63], was not mentioned in any of the included studies. Furthermore, surgeons’ advice is a main reason for patients to refrain from sports participation after THA [15, 63], but none of the included studies adjusted for this. Compared with the 1999 Hip Society recommendations for athletic activity after THA [64], subsequent expert opinion surveys found an increasing tolerance for and acceptance of granting patients permission to return to higher-impact activities, such as downhill skiing or ice skating [14, 65, 66]. Interestingly, Swanson et al. [65] found that high-volume hip surgeons were more liberal in their recommendations, indicating that, as experience with THA and RTS grows, orthopedic surgeons become less cautious. Thus, a trend of allowing more sports activities after THA can be observed over the past 2 decades. Although our data suggest that patients do engage in intermediate- and high-impact sports activities, we observed a shift from high-impact sports activities to low-impact sports activities. From a surgeon’s point of view, this may seem desirable, but it is important to discuss this with the patient before surgery. High-impact sports activities are known to negatively influence THA durability [67]. However, as some of the included studies have shown, a limited proportion of patients do participate in high-impact activities such as tennis, running, and judo. For many of these patients, the possibility of continuing their high-impact activities is the main reason for surgery. It appears that the experience level of patients, which was high in all patients returning to high-impact activities, influences the possibility of RTS. Thus, despite the increased risk of mechanical failure, a return to high-impact activities should not be completely ruled out, especially in experienced patients. The 15-year survival rate in highly active patients is reported to be approximately 80% for bearings that were used 20 years ago [68]. The risk of dislocation and fractures is low. To address the needs of active patients, surgeons can use bearings with low wear rates and femoral heads up to 36 mm in diameter [68]. Future studies are needed to identify the thresholds for physical activity in patients undergoing hip arthroplasty, above which the negative effects in terms of prosthesis wear exceed the positive effects in terms of general health improvement and patient satisfaction [15, 17].

#### Prognostic Factors for Return to Work

This systematic review is one of the first studies to systematically summarize the effects of confounding factors on RTW [11, 69] and the first to summarize the effects of confounding factors on RTS. These findings provide additional guidance for the orthopedic surgeon and the patient in preoperative counseling and discussing the probability of RTW and RTS. Regarding RTW, a previous literature review identified two studies of confounding factors influencing RTW after THA, both of which found that preoperative sick leave was associated with lower RTW [70]. Another review identified 15 studies describing beneficial or limiting factors for RTW after THA but did not summarize these findings [11]. Our systematic review identified 22 studies reporting on confounding factors for RTW. Preoperative sick leave appears to be a consistent prognostic factor for RTW. All studies that included preoperative sick leave in the analysis found lower RTW for patients who were absent from work preoperatively, which is in line with findings in patients undergoing knee arthroplasty [24, 71, 72]. Furthermore, a higher preoperative workload was associated with lower RTW in most studies. Similarly, a higher level of education, which results in more white-collar jobs and thus a lower physical workload, was associated with higher RTW. Factors such as collecting a preoperative disability pension or disability insurance, job satisfaction, employment versus self-employment, and THA approach, might be associated with RTW. However, only a limited number of studies investigated these factors, and more research is needed to verify this. To this end, future studies of RTW in THA patients should collect and discuss these factors.

### Limitations

A limitation of the present analysis is the low availability of high-quality, prospective studies. Most studies had a retrospective design, thus increasing the risk of recall bias. Also, methodological quality was rated as moderate or low in most studies. Furthermore, no validated questionnaires for the assessment of RTS and RTW exist, which hampers comparisons between studies. The development of a core outcome set will allow for more reliable and valid collection of patient data on work and sports participation before and after THA [73].

## Conclusion

Most patients were able to RTS and RTW after THA within a timeframe of 28 and 17 weeks, respectively. Participation in high-impact sports activities is less likely but not impossible. Furthermore, RTW with a high workload or after prolonged preoperative sick leave is less likely. For the increasingly younger THA population, this is valuable information that can be used by the orthopedic surgeon and the patient in the preoperative shared decision-making process.

## Electronic supplementary material

Below is the link to the electronic supplementary material.

Supplementary material 1 (XLSX 13 kb)

Supplementary material 2 (DOC 28 kb)

Supplementary material 3 (DOC 28 kb)
