# Supplementary material for: The Effect of Total Hip Arthroplasty on Sports and Work Participation: A Systematic Review and Meta-Analysis
Source: Sports Med. 2018 Apr 24;48(7):1695–726. doi: 10.1007/s40279-018-0924-2 (PMC5999146; doi:10.1007/s40279-018-0924-2)
Supplement: Supplementary file 2 — Supplementary material 2 (DOC 28 kb) [file 40279_2018_924_MOESM2_ESM.doc]

**Electronic Supplementary Material Appendix S2.** Newcastle – Ottawa Scale for the assessment of methodological quality

**NEWCASTLE - OTTAWA QUALITY ASSESSMENT SCALE**

**COHORT STUDIES**

Note: A study can be awarded a maximum of one star for each numbered item within the Selection and Outcome categories. A maximum of two stars can be given for Comparability

**Selection**

1) Representativeness of the exposed cohort

a) truly representative of the average THA patients with (end stage) OA in the working age (average age <65 years) in the community **🟑**

b) somewhat representative of the average THA patients with (end stage) OA in the working age in the community **🟑**

c) selected group of patients with trauma, with infections, athletes, or selection based on activity scores (e.g. UCLA score)

d) no description of the derivation of the cohort

2) Selection of the non exposed cohort

a) drawn from the same community as the exposed cohort **🟑**

b) drawn from a different source

c) no description of the derivation of the non exposed cohort

3) Ascertainment of exposure

a) secure record (eg surgical records) **🟑**

b) structured interview **🟑**

c) written self report

d) no description

4) Demonstration that outcome of interest was not present at start of study

a) yes **🟑**

b) no

**Comparability**

1) Comparability of cohorts on the basis of the design or analysis

a) study controls for pre-op work or sport status (select the most important factor) **🟑**

b) study controls for any additional factor: age, sex, BMI, motivation, surgeons advice, workload, satisfaction with job **🟑** (This criteria could be modified to indicate specific control for a second important factor.)

**Outcome**

1) Assessment of outcome

a) independent blind assessment **🟑**

b) record linkage **🟑**

c) self report

d) no description

2) Was follow-up long enough for outcomes to occur

a) yes ≥1 year (select an adequate follow up period for outcome of interest) **🟑**

b) no

3) Adequacy of follow up of cohorts

a) complete follow up - all subjects accounted for **🟑**

b) subjects lost to follow up unlikely to introduce bias - small number lost - > 80% (select an adequate %) follow up, or description provided of those lost) **🟑**

c) follow up rate < 80% (select an adequate %) and no description of those lost

d) no statement
