# Supplementary material for: The Effect of Total Hip Arthroplasty on Sports and Work Participation: A Systematic Review and Meta-Analysis
Source: Sports Med. 2018 Apr 24;48(7):1695–726. doi: 10.1007/s40279-018-0924-2 (PMC5999146; doi:10.1007/s40279-018-0924-2)
Supplement: Supplementary file 3 — Supplementary material 3 (DOC 28 kb) [file 40279_2018_924_MOESM3_ESM.doc]

**Electronic Supplementary Material Appendix S3.** Levels of impact on hip joint of different types of sports participation ^a^

| **Low-impact** | **Intermediate-impact** | **High-impact** |
| --- | --- | --- |
| Stationary cycling  Golf  Cross-country skiing  Swimming  Walking  Dancing  Water aerobics  Potentially Low:  Bowling  Fencing  Rowing  Isokinetic weight lifting  Sailing  Speed walking  Table tennis  Bicycling | Free weight lifting  Hiking  Horseback riding  Ice skating  Rock climbing  Low-impact aerobics  Doubles tennis  In-line skating  Downhill skiing | Baseball / softball  Basketball  Volleyball  Football  Handball  Racquetball  Jogging/ running  Lacrosse  Soccer  Singles tennis  Water skiing  Karate |

^a^According to Vail et al [26].
